# *Candidozyma auris* (formerly *Candida auris*): A P6 medicine framework integrating genomics, surveillance and the prevention of multidrug-resistant fungal threats

**DOI:** 10.3892/mi.2026.343

**Published:** 2026-10-05

**Authors:** Styliani Geronikolou, George P. Chrousos

**Affiliations:** 1 University Research Institute of Maternal and Child Health and Precision Medicine and UNESCO Chair on Adolescent Health Care, National and Kapodistrian University of Athens, 11527 Athens, Greece; 2 Hellenic Pasteur Institute, 11521 Athens, Greece

**Keywords:** *Candidozyma auris*, healthcare-associated infections, genomic epidemiology, antifungal stewardship, infection prevention and control, clinical microbiology, public health governance, multidrug resistance

## Abstract

Healthcare-associated infections are major drivers of global morbidity; yet, fungal threats remain under-characterized. *Candidozyma auris* (*C. auris*; formerly known as *Candida auris*) has emerged as a critical public health challenge due to its extensive multidrug-resistant profile, environmental resilience and high transmissibility. Its resistance to major antifungal classes, including azoles, polyenes and echinocandins, severely limits therapeutic options and exposes the limitations of fragmented, discipline-specific healthcare responses. The present conceptual commentary proposes that the persistence of *C. auris* highlights limitations in fragmented, discipline-specific healthcare responses. Herein, the predictive, preventive, personalized, participatory, psycho-cognitive and public (P6) model of medicine is proposed as a comprehensive framework to move beyond reactive containment. By repositioning clinical microbiology as the entry point for surveillance through early, accurate diagnosis, the P6 model integrates genomic epidemiology, antifungal stewardship and behavioral science to support earlier detection of emerging threats and inform timely clinical, infection prevention and control, and public health responses. The emergence of *C. auris* provides a practical test of the ability of healthcare systems to integrate microbiological intelligence, prevention strategies and public-health governance by replacing delayed laboratory identification, fragmented surveillance, or poor data sharing. Addressing its high resistance and rapid spread requires an integrative approach that leverages advanced analytics and public health governance. The P6 framework provides a scalable strategy to strengthen health systems against both current and future multidrug-resistant fungal pathogens.

## Infections, *Candidozyma auris* and the P6 model

Healthcare-associated infections (HAIs) remain among the most frequent and costly adverse events worldwide, contributing substantially to preventable morbidity, mortality and health-system strain. Although tens of millions of patients are estimated to acquire infections annually through healthcare exposure, the true magnitude remains uncertain owing to heterogeneous surveillance systems, variable diagnostic capacity and inconsistent reporting standards (1). These limitations disproportionately affect pathogens that rely on specialized laboratory identification and antifungal susceptibility testing, allowing critical threats to remain under-recognized until outbreaks occur.

Within this under-characterized landscape, fungal infections are persistently under-recognized despite their disproportionate contribution to global mortality. Invasive fungal diseases are estimated to affect 6 million individuals annually, resulting in ~3-4 million deaths, either directly or in the context of severe underlying illness. These figures, although constrained by methodological uncertainty, underscore an escalating public health challenge exacerbated by delayed diagnosis, limited resistance surveillance and the insufficient integration of fungal pathogens into routine HAI monitoring frameworks (2).

In 2022, the World Health Organization (WHO) included *Candidozyma auris* (formerly known as *Candida auris*, henceforth referred to *C. auris*) in its first Global Priority List of Fungal Pathogens of critical concern. This designation reflects a convergence of high-risk characteristics: Multidrug resistance, environmental persistence, healthcare-associated transmission and a marked capacity to cause sustained outbreaks across diverse clinical settings (3-5). Notably, *C. auris* has emerged at the interface of clinical microbiology, antifungal stewardship, and infection prevention and control (IPC), exposing gaps created by fragmented, discipline-specific responses.

The clinical challenge posed by *C. auris* is rooted in its complex and evolving resistome. Resistance to azoles is commonly associated with mutations in *ERG11* and the overexpression of efflux pumps, while echinocandin resistance is primarily mediated by hotspot mutations in *FKS1* (6,7). Although resistance to polyenes appears less frequent, reduced susceptibility has been documented and remains incompletely understood. These mechanisms significantly constrain therapeutic options, particularly in critically ill patients. They also underscore the need for timely species identification, antifungal susceptibility testing, and resistance surveillance to inform clinical management and stewardship strategies (4,8).

Beyond genetic resistance, *C. auris* exhibits marked environmental resilience. It tolerates heat, osmotic stress and several commonly used disinfectants, and readily forms biofilms on medical devices and hospital surfaces. These properties facilitate prolonged environmental persistence and recurrent transmission, particularly in intensive care units and long-term care facilities. Genomic analyses have identified at least five major clades with distinct geographic distributions and resistance profiles, supporting the hypothesis of multiple, near-simultaneous emergences rather than dissemination from a single source (7-10). From an operational perspective, these findings highlight the importance of linking genomic epidemiology with local laboratory surveillance and IPC interventions. Despite growing recognition, the management of *C. auris* infections remains largely reactive. Current approaches emphasize case detection, patient isolation, cohorting and intensified environmental cleaning after colonization or infection has occurred. While essential, these measures are often implemented at a late stage, are resource-intensive, and may fail to prevent recurrent transmission when laboratory detection, resistance data and IPC decision-making are insufficiently integrated (11,12).

More fundamentally, the emergence of *C. auris* exposes structural limitations in healthcare systems that prioritize individual-level treatment over anticipatory, system-wide prevention (13).

Surveillance programs frequently focus on bacterial pathogens, with fungal organisms underrepresented in routine reporting (14). Data sharing between institutions may be inconsistent, and laboratory capacity for advanced diagnostics or genomic sequencing remains uneven across regions. These gaps undermine early detection, delay coordinated responses, and allow outbreaks to expand before comprehensive containment measures are deployed.

Addressing these limitations requires more than incremental improvements in diagnostics or environmental cleaning. It demands a framework capable of linking molecular insight, clinical decision-making, behavioral engagement and public-health coordination. The predictive, preventive, personalized, participatory, psycho-cognitive and public (P6) model of medicine provides such an integrative approach. Extending beyond traditional precision medicine, which often centers primarily on individualized therapy, P6 explicitly incorporates population health, behavioral science and governance into a unified model. In doing so, it positions clinical microbiology, not merely as a diagnostic service, but as a sentinel discipline within healthcare systems (15).

Applied to *C. auris*, the P6 model enables predictive and personalized care using clinical, microbiological and genomic data to identify high-risk patients and wards before transmission escalates. The preventive component emphasizes proactive laboratory screening policies, environmental monitoring and early IPC engagement, rather than reliance on post hoc containment. Personalized care integrates antifungal susceptibility data and patient-specific risk factors to optimize therapy, while limiting unnecessary antifungal exposure (Fig. 1).

The participatory dimension recognizes that effective containment depends on the engagement of healthcare workers, patients and caregivers. Education on transmission dynamics, adherence to IPC protocols, and transparent communication during outbreaks are critical for sustaining compliance. The psycho-cognitive element addresses often-overlooked factors, such as stigma associated with isolation, alert fatigue among staff and psychological stress during prolonged outbreaks, factors that can erode adherence and compromise IPC effectiveness. Finally, the public dimension situates *C. auris* within broader surveillance and governance systems, promoting harmonized reporting standards, cross-institutional data sharing, and alignment between antifungal resistance surveillance and IPC programs at regional and national levels (Fig. 1). The core conceptual shifts required to move from these traditional, reactive approaches to a P6-integrated paradigm are summarized in Table I.

Artificial intelligence and advanced analytics can strengthen the P6 framework by integrating electronic health records, laboratory information systems and genomic databases to identify high-risk patients and anticipate outbreak hotspots, transmission clusters or resistance trends. However, implementation remains uneven and requires careful governance-accompanied by robust governance, interoperability standards and safeguards to ensure responsible data use and equitable access to advanced diagnostics (16). Notably, adopting an integrative framework does not diminish the importance of established IPC measures. Rather, it strengthens them by embedding these measures within a predictive and system-oriented structure. Clinical microbiology laboratories, with their access to diagnostic, susceptibility and genomic data, are uniquely positioned to generate the microbiological intelligence that triggers integrated responses. By rapidly communicating laboratory findings to clinicians, epidemiologists, infection prevention and control teams, antifungal stewardship programs, public health inspectors and public health authorities, laboratories transform diagnostic data into actionable intelligence that supports coordinated surveillance, investigation and control.

Finally, the public dimension situates *C. auris* within broader surveillance and governance systems, promoting harmonized reporting standards, cross-institutional data sharing, and alignment between antifungal resistance surveillance and IPC programs at regional and national levels (3,11). This dimension also recognizes the complementary responsibilities of clinical microbiologists, epidemiologists, public health inspectors, IPC professionals and public health authorities (12). Clinical microbiology provides the microbiological intelligence that initiates surveillance and response activities, epidemiologists investigate transmission dynamics and analyze surveillance data, public health inspectors evaluate environmental and regulatory compliance within healthcare facilities (17), while public health authorities coordinate surveillance systems, policy implementation, and population-level responses (3,11,12).

## Operationalizing the P6 framework for the prevention and control of *C. auris*

Although the P6 framework is conceptual in nature, its principles can be translated into practical health-system actions. An integrated response can be organized across three complementary phases: Pre-emptive prevention, targeted clinical management and coordinated systems-level governance.

*Phase 1: Predictive and preventive response*. The predictive and preventive dimensions aim to shift the management of *C. auris* from outbreak response to early risk identification. Hospitals may implement targeted screening strategies for high-risk populations, including patients transferred from healthcare facilities with known transmission, individuals requiring intensive care, and those with prolonged healthcare exposure. During outbreaks or in high-risk settings, targeted environmental assessment may complement patient screening, particularly given the ability of *C. auris* to persist on surfaces and medical equipment (18).

Where infrastructure permits, the integration of electronic health records, laboratory information systems, and genomic surveillance platforms may support identification of transmission patterns and emerging outbreak clusters. However, these approaches should complement, rather than replace, foundational requirements such as diagnostic capacity, surveillance infrastructure, and effective IPC programs.

### Phase 2: Personalized and psycho-cognitive response

Once *C. auris* is detected, management should integrate biological characteristics of the organism with patient-specific and organizational factors. Rapid species identification, antifungal susceptibility testing and genomic analysis where available can support personalized therapeutic decisions and identification of resistance-associated mechanisms, including alterations involving ERG11 and FKS1. Within this phase, antifungal stewardship programs provide the clinical governance mechanism to translate microbiological findings into appropriate treatment decisions, including the optimization of antifungal selection, dosing, duration, and the avoidance of unnecessary exposure that may contribute to resistance (19).

The psycho-cognitive dimension recognizes that successful outbreak control depends not only on microbiological interventions, but also on human behavior. Prolonged isolation precautions may contribute to patient distress and stigma, while repeated alerts and changing IPC requirements may contribute to staff fatigue. Addressing these factors through communication strategies and staff support is essential for maintaining adherence (20).

### Phase 3

The participatory and public dimensions extend management beyond individual institutions and emphasize multidisciplinary collaboration across clinical, laboratory and public health systems (21). Clinical microbiology laboratories function as the triggering hub by providing timely organism identification, antifungal susceptibility testing, and genomic intelligence that initiate surveillance and response activities. Effective responses require structured communication pathways through which laboratory findings are rapidly shared with clinicians, infection prevention and control teams, epidemiologists, antifungal stewardship programs, public health inspectors and public health authorities.

Epidemiologists analyze surveillance data, investigate transmission dynamics, identify outbreak clusters, and perform risk assessments that guide evidence-based public health interventions (17). Public health inspectors contribute through healthcare facility inspections, environmental investigations, assessment of infection prevention and control compliance, and implementation of regulatory measures where appropriate (17). Public health authorities coordinate surveillance systems, harmonize reporting standards, facilitate cross-institutional data sharing, and oversee policy implementation and outbreak response. Together, these complementary functions strengthen early detection, coordinated containment, and health-system resilience against multidrug-resistant fungal pathogens.

The contribution of the present study is the adaptation of the P6 model from an individual-centered precision medicine paradigm toward a health-system framework for managing multidrug-resistant fungal threats, with clinical microbiology positioned as the triggering source of microbiological intelligence that informs clinical decision-making, epidemiological surveillance, infection prevention and control, and public health action within an integrated health-system framework.

The continued spread of HAIs reflects the failures of integration across microbiological diagnostics, antifungal resistance surveillance, clinical management, and infection prevention and control. *C. auris* represents more than an emerging multidrug-resistant pathogen; it is a stress test for siloed approaches of modern healthcare systems, exemplified by the diagnostic lags and unintegrated electronic health records that allow undetected colonization to spread during inter-facility patient transfers. Positioning clinical microbiology as the triggering node for microbiological intelligence within an integrative framework enables the earlier recognition of emerging threats and provides the evidence base for coordinated action by clinicians, infection prevention and control teams, epidemiologists, public health inspectors and public health authorities. Such an approach strengthens preparedness not only for *C. auris*, but also for future healthcare-associated fungal threats.

## Figures and Tables

**Figure 1:**
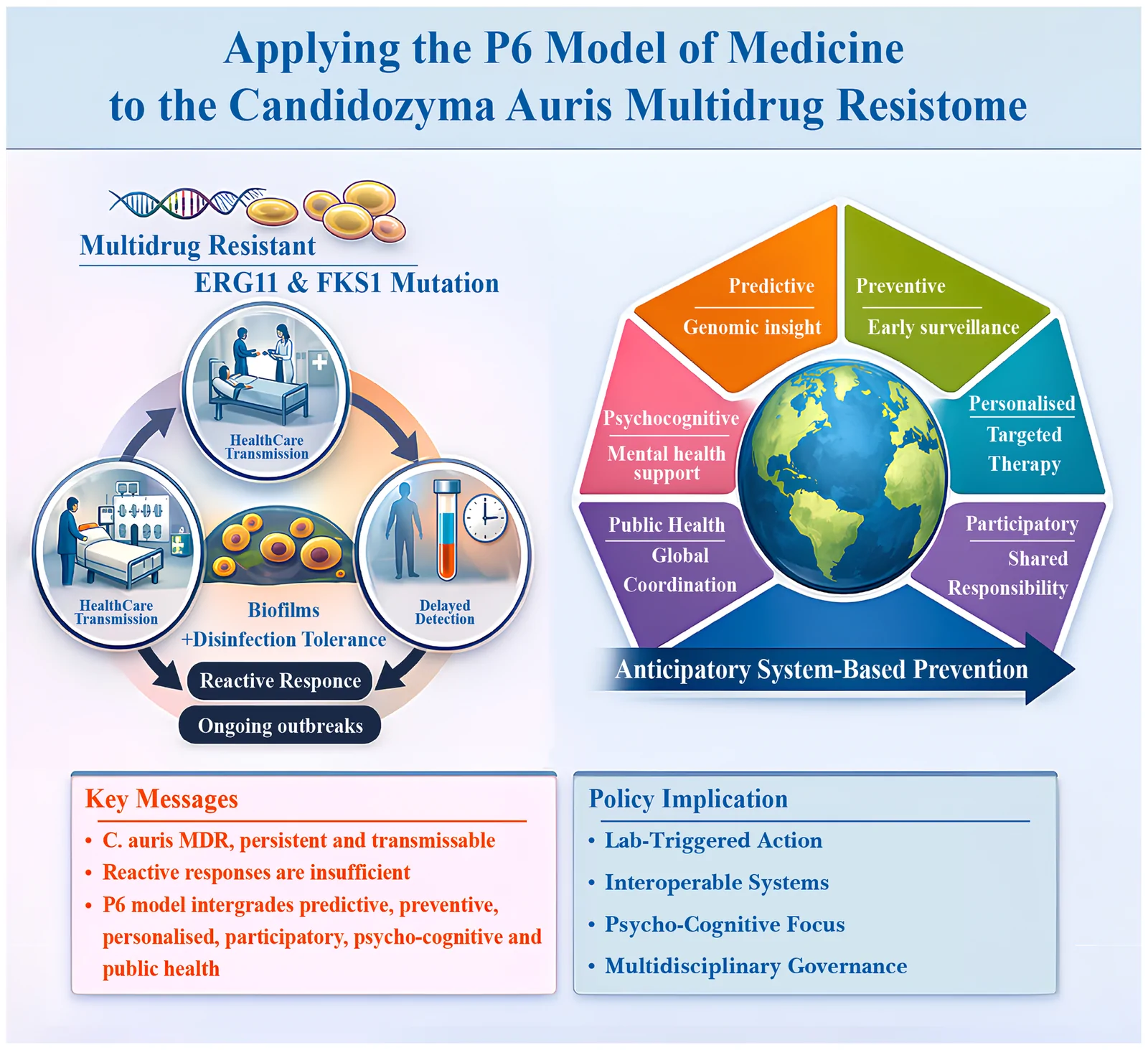
Applying the P6 medicine framework to the prevention and control of multidrug-resistant *Candidozyma (Candida) auris*. The left panel illustrates the limitations of conventional reactive approaches, in which antifungal resistance associated with *ERG11* and *FKS1* mutations, biofilm formation, disinfectant tolerance, healthcare transmission, and delayed detection contribute to persistent outbreaks. The right panel presents the P6 medicine framework, integrating predictive (genomic insight), preventive (early surveillance), personalized (targeted therapy), participatory (shared responsibility), psycho-cognitive (mental health and behavioral support), and public health (global coordination) principles to enable anticipatory, systems-based prevention. Together, these complementary strategies support genomic surveillance, precision diagnosis and treatment, infection prevention and control, stakeholder engagement, and multidisciplinary public health governance. Specifically, policy implementation relies on lab-triggered system action, interoperable genomic and electronic health record data sharing, psycho-cognitive support to address staff alert fatigue and patient isolation stigma, and coordinated oversight uniting public health authorities, inspectors, and infection control teams to reduce transmission and improve preparedness against multidrug-resistant *C. auris*. MDR, multidrug-resistant; IPC, infection prevention and control; EHR, electronic health record.

**Table I:** Key conceptual shifts introduced by the P6 framework for *C. auris* surveillance, prevention and control.

Domain	Reactive approach	P6-integrated approach
Trigger for action	Detection of clinical infection or outbreak	Early microbiological intelligence, predictive risk assessment, surveillance signals, and genomic detection before widespread transmission
Role of microbiology	Diagnostic service provider	Triggering source of actionable microbiological intelligence that initiates multidisciplinary surveillance, clinical decision-making, and IPC interventions
Surveillance model	Fragmented laboratory, clinical, and public health activities	Integrated surveillance linking clinical microbiology, epidemiology, IPC, public health inspection, stewardship, and governance
Data utilization	Separate laboratory and clinical datasets	Integrated microbiological, genomic, epidemiological, environmental, behavioral and clinical intelligence
Primary focus	Containment and treatment after detection	Prevention, prediction, personalized management and coordinated multidisciplinary response
Psycho-cognitive dimension	Emphasis on protocol compliance	Inclusion of behavioral, psychological, and organizational considerations-alert fatigue, isolation-related stigma, and communication- to improve adherence, communication, resilience, and sustained IPC performance
Participatory and public dimension	Limited stakeholder engagement and institution-specific response	Active participation of healthcare workers, patients, caregivers, and coordinated action through surveillance, public health inspection and governance

## Data Availability

Not applicable.
